# The Roles of Happiness Motives in Cognitive Reappraisal and Expressive Suppression: A Longitudinal Study in Emerging Adults

**DOI:** 10.3390/bs16030312

**Published:** 2026-02-24

**Authors:** Wenjie Li, Kairong Yang, Feng Kong

**Affiliations:** 1 School of Psychology, Shaanxi Normal University, Xi’an 710062, China; lwj10011001@163.com; 2 Department of Psychology and Cognitive Sciences, Tsinghua University, Beijing 100084, China; 3 Department of Health and Science, Xi’an Physical Education University, Xi’an 710062, China

**Keywords:** hedonic and eudaimonic motives, emotion regulation, emerging adults, longitudinal study

## Abstract

It has been hypothesized that happiness motives (including hedonic and eudaimonic motives) are associated with emotion regulation, which could further influence individuals’ well-being. However, less is known about the longitudinal effects of these motives on emotion regulation in emerging adults. To address this gap, the present study employed a two-wave longitudinal design with a six-month interval among a sample of 287 emerging adults (18–26 years; *M* = 20.23 and *SD* = 1.60). The findings showed that hedonic and eudaimonic motives were significantly associated with cognitive reappraisal, whereas no significant associations were observed with expressive suppression. Importantly, eudaimonic motives positively predicted cognitive reappraisal six months later, but hedonic motives did not, whereas neither motive significantly predicted expressive suppression. This association remained significant when age and gender were included as covariates. These findings extend the research on happiness motives and emotion regulation by providing longitudinal insights and have important practical implications for practitioners in terms of reinforcing eudaimonic motives to better utilize cognitive reappraisal strategies.

## 1. Introduction

Emotion regulation is generally defined as the processes by which individuals influence their emotions, including when and how they experience and express them (Gross, 1998; Maloney et al., 2023). It is widely accepted that a central function of emotion regulation is to shape emotional experiences in ways that support psychological well-being and adaptive functioning (Larsen, 2000; Tamir, 2009; Zhi & Derakhshan, 2024). Moreover, a growing body of evidence suggests that more effective emotion regulation is associated with higher well-being (Gubler et al., 2021; H. Wang et al., 2023), better academic performance (Nadeem et al., 2023), and lower depression (Diedrich et al., 2017; Depoorter et al., 2025). To achieve these adaptive outcomes, individuals employ a range of strategies to modulate their emotional experiences and expressions (Paley & Hajal, 2022).

According to the process model of emotion regulation, strategies can be classified based on the stage at which they intervene in the emotion-generative process (Gross, 2015; De Neve et al., 2023). Specifically, antecedent-focused strategies are applied before an emotional response is fully activated, whereas response-focused strategies are implemented after emotional responses have already been generated. Among these strategies, cognitive reappraisal and expressive suppression are the two most extensively studied forms of emotion regulation (Greenaway et al., 2025; Sheppes, 2020). As an antecedent-focused strategy, cognitive reappraisal regulates emotional responses by reframing the meaning of an emotion-provoking situation. In contrast, expressive suppression functions as a response-focused strategy that involves inhibiting ongoing emotion-expressive behavior (Preece & Gross, 2025; Gross & John, 2003).

Previous research has demonstrated that individuals’ choices of emotion regulation strategies are influenced by multiple factors, including emotion-related beliefs, contextual affordances, mindfulness, and, critically, motivational orientations (Kneeland et al., 2016; Raugh & Strauss, 2024; Rowland et al., 2023; Suri et al., 2018; Tamir, 2016). Among these factors, motivation plays a particularly central role, as it guides individuals’ intentional behavior and decision-making, including how they evaluate the costs and benefits of different emotion regulation strategies. Hedonia and eudaimonia are considered fundamental driving forces behind individuals’ behavioral choices (Huta, 2016; Pearce & Huta, 2023). Consequently, happiness motives (i.e., hedonic motives and eudaimonic motives) may play a crucial role in shaping how individuals regulate their emotions. Hedonic motives refer to pursuing good feelings (e.g., pleasure and relaxation), while eudaimonic motives are characterized as striving to live life to one’s potential and having goals larger than oneself (Huta & Ryan, 2010; Huta & Pearce, 2025). Accumulating research has delineated the distinct and overlapping roles of these two motives in the context of emotion regulation and well-being. While both motives can contribute to well-being, eudaimonic pursuits are often more strongly linked to sustained psychological flourishing and integrated self-functioning (Curren & Park, 2024). Interestingly, this pattern does not hold in all settings. In specific achievement contexts such as second language learning, hedonic orientations showed a significant positive correlation with trait emotional intelligence and regulation capacity, whereas eudaimonic orientations demonstrated a positive but non-significant association (Rad & Hashemian, 2023). Furthermore, the motivational context behind the use of a specific strategy can alter its outcomes. For instance, the typically negative impact of expressive suppression on psychological well-being can be attenuated when employed for instrumental goals (e.g., to maintain social harmony) or for contra-hedonic goals (Han & Kim, 2024). This underscores that the adaptiveness of an emotion regulation strategy depends not only on its type but also on the motive underlying its use.

Previous studies provided initial evidence that hedonic and eudaimonic motives are differentially associated with emotion regulation strategies (Ortner et al., 2018; Rad & Hashemian, 2023). Notably, recent research on long-term emotional goals—which often reflect eudaimonic aims—found that greater attention to such goals during negative emotions is associated with higher well-being and facilitates the use of more engagement-focused strategies like reappraisal (Veilleux et al., 2024). Given that effective emotion regulation, such as reappraisal, is considered a vital predictor of well-being and adaptive functioning (Côté et al., 2010; Gross, 2014; Verzeletti et al., 2016), a deeper exploration of how happiness motives shape emotion regulation could elucidate the motivational pathways to psychological health.

Drawing on the process model of emotion regulation (Gross, 2015; De Neve et al., 2023), cognitive reappraisal and expressive suppression differ in their timing and regulatory demands, leading to differential associations with happiness motives. Cognitive reappraisal acts early in the emotion-generative process and may be especially well aligned with motives that emphasize sustained emotional benefits. Consistent with this, Ortner et al. (2022) demonstrated that long-term regulatory motives predicted the use of cognitively oriented strategies beyond the influence of hedonic and instrumental motives. Moreover, in daily life, individuals tend to employ cognitive reappraisal more frequently when motivated by both hedonic (e.g., to feel better) and instrumental goals (English et al., 2017). Further evidence supports this, indicating that adolescents with stronger pro-hedonic motives report greater use of reappraisal, while those with stronger contra-hedonic motives tend to rely more on suppression (Shao et al., 2025). Conversely, suppression is often more closely linked to motives involving impression management or enduring short-term discomfort for longer-term instrumental gains (English et al., 2017; Shao et al., 2025). Thus, we hypothesized that both hedonic and eudaimonic motives might be positively related to cognitive reappraisal, whereas only eudaimonic motives might be associated with expressive suppression.

The theoretical rationale for focusing specifically on cognitive reappraisal and expressive suppression in relation to happiness motives is threefold. First, they represent prototypical of antecedent-focused and response-focused strategies, respectively, offering a clear theoretical contrast in terms of timing and cognitive demands (Gross, 2015; De Neve et al., 2023), allowing for a clear theoretical contrast. Second, as outlined above, empirical evidence consistently links these two strategies to different motivational profiles in daily life and cross-sectional studies (English et al., 2017; Ortner et al., 2018; Shao et al., 2025). Third, although other antecedent-focused strategies such as distraction exist, they are typically employed to manage high-intensity immediate emotions and are more tightly linked to immediate hedonic relief rather than the broader construct of eudaimonic motives (Sheppes & Gross, 2011; Sheppes et al., 2014; Zhang et al., 2022). By comparison, reappraisal involves cognitive reframing, making it theoretically more relevant to eudaimonic pursuits that involve meaning-making, authenticity, and alignment with values—concepts central to eudaimonia and integrative emotion regulation (Curren & Park, 2024).

Furthermore, we focused on emerging adulthood as it is a crucial developmental stage characterized by various developmental challenges (Ojala, 2023; Roisman et al., 2004). During this period, while expressed anger decreases slowly, while depression may still increase until age thirty (Galambos et al., 2006; Hemberg et al., 2022). This suggests that emotional stability persists during emerging adulthood (Zimmermann & Iwanski, 2014; d’Huart et al., 2022). To better navigate these developmental challenges, individuals need to engage in meaningful activities and master learning tasks, especially the ability to regulate their emotions (Güçlü et al., 2022; Tammilehto et al., 2021). Emerging adults typically engage in a broader array of activities than other age groups, as they face fewer constraints from established social roles (Arnett, 2000; Ojala, 2023). Moreover, the activities individuals participate in may impact their well-being and mental health (Cruyt et al., 2021; Hooker et al., 2020; Matud et al., 2023). Therefore, it is essential to assess happiness motives in emerging adults and their effects on emotion regulation, which could provide a theoretical basis for intervention aimed at improving their well-being and mental health.

To date, existing research has provided important initial evidence that hedonic and eudaimonic motives are linked to emotion regulation strategies, both at the trait level (Ortner et al., 2018; Rad & Hashemian, 2023) and at the momentary level in daily life (Ortner et al., 2022). However, it remains unclear whether happiness motives prospectively shape emotion regulation patterns over time, particularly during emerging adulthood when motivational orientations and regulatory habits are still developing. To address this gap, the present study examined the prospective effects of happiness motives on emotion regulation during emerging adulthood using a two-wave longitudinal framework. Given that age and gender have been identified as relevant factors associated with happiness motives (Brakus et al., 2022; Gentzler et al., 2021; Matranga et al., 2020) and emotion regulation (Coats & Blanchard-Fields, 2008; Isaacowitz, 2022; Nolen-Hoeksema, 2012; Zimmermann & Iwanski, 2014), we controlled for these variables in our analyses. Based on the theoretical framework and empirical gaps outlined above, we hypothesized that both hedonic and eudaimonic motives would be positively related to cognitive reappraisal six months later, whereas only eudaimonic motives would be positively associated with expressive suppression. We expected these patterns to remain robust even after controlling for age and gender.

## 2. Materials and Methods

### 2.1. Subjects and Procedure

The sample was obtained from a university in China. Four hundred and thirty-two students volunteered to engage in this study (Time 1). After providing informed consent and understanding the purpose and the procedure of our study through written and verbal instructions, participants completed the measure of hedonic motives, eudaimonic motives, and emotion regulation through an online website (https://www.wjx.cn/). Follow-up data collection was conducted after six months (Time 2), and the participants were asked to fill out the same measure as Time 1. Finally, a total of 287 participants engaged in two measurement waves, and the attrition rate was 33.6%. According to the power analysis calculated by G*Power 3.0, we needed at least 193 individuals for a small correlation (*r* = 0.20, α = 0.05, 1 − *β* = 0.95) (Faul et al., 2007). Thus, the sample size in this study was sufficient. The sample includes 38 males and 249 females aged 18–26 years (*M* = 20.23, *SD* = 1.60). Ethics approval was granted by the local university.

### 2.2. Materials

Hedonic and Eudaimonic Motives: The revised version of the Hedonic and Eudaimonic Motives for Activities Scale (HEMA-R; Huta, 2016) was utilized. Participants responded to a 7-point Likert scale ranging from 1 (not at all) to 7 (very much), indicating the extent to which they commonly engage in activities with hedonic or eudaimonic purposes. The scale consists of two 5-item subscales: hedonic motives (e.g., “Seeking pleasure”, α_T1_ = 0.842, α_T2_ = 0.784) and eudaimonic motives (e.g., “Seeking to pursue excellence or a personal ideal”, α_T1_ = 0.894, α_T2_ = 0.899). The HEMA-R has shown good indices of reliability and validity in the Chinese version (Jia et al., 2021; Li et al., 2021; Li et al., 2022).

Emotion Regulation. The Emotion Regulation Questionnaire (ERQ; Gross & John, 2003) asks participants to evaluate the degree they agreed with the items on a 7-point Likert scale ranging from 1 (strongly disagree) to 7 (strongly agree). The scale contains two subscales, cognitive reappraisal includes six items (α_T1_ = 0.856, α_T2_ = 0.887), like “When I want to feel more positive emotion (such as joy or amusement), I change what I’m thinking about”, while expressive suppression contains four items (α_T1_ = 0.808, α_T2_ = 0.837), for example, “When I am feeling negative emotions, I make sure not to express them”. It has been demonstrated that the scale has acceptable psychometric properties in Chinese (Zhao & Zhao, 2015).

### 2.3. Data Analyses

Firstly, to test for common method bias that could potentially bias our results, we carried out Harman’s single-factor analysis (Podsakoff et al., 2003; Qu, 2025). Our results indicated that the variance explanation rate of a single factor was 22.91% (<40%). Thus, the common method bias should not be an issue in the present study.

In addition, we chose maximum likelihood estimation with robust standard errors (MLR) to analyze data in Mplus 7.4. Preliminarily, the confirmatory factor analysis (CFA) model was tested, which includes all variables utilizing latent factor scores and the regression paths we were interested in were not modeled. All missing data and outliers were replaced with 999 in further analyses so that they would not be included in analyses. We used some indices to examine the applicability of models: Akaike Information Criterion (AIC), and Bayesian Information Criterion (BIC), standardized root mean square residual (SRMR), root mean square error of approximation (RMSEA), comparative fit index (CFI) (Hu & Bentler, 1999). Accordingly, values of RMSEA <0.08, SRMR <0.10, and CFI >0.90 were evaluated as indicators of good fit. Smaller values of AIC and BIC mean a better fit.

Then, the measurement reliability across time was tested using the metric and scalar invariance (Selig & Little, 2012; Specker, 2024). More specifically, a metric invariance model was examined with factor loadings constrained across two time points to be equal. In addition, a scalar invariance model was tested with factor loadings and intercepts across two time points were constrained. Changes in CFI values (∆CFI) less than 0.01 were considered indicative of measurement invariance (Cheung & Rensvold, 2002; Jiang et al., 2023).

Subsequently, we constructed a predictive model to empirically test our hypotheses regarding the longitudinal influence of happiness motives on emotion regulation. In this structural model, directional paths were specified from both hedonic and eudaimonic motives to cognitive reappraisal and expressive suppression. Furthermore, to verify the hypothesized robustness of these associations, age and gender were included as covariates to control for their potential confounding roles.

## 3. Results

### 3.1. Descriptive Analysis

Table 1 shows descriptive statistics and correlations among happiness motives and emotion regulation. For expressive suppression, only expressive suppression at T2 was positively related to expressive suppression at T1 and negatively linked to cognitive reappraisal at T1; the rest of the variables were not significantly related to expressive suppression. Except for expressive suppression, all the other variables were significantly associated with each other.

### 3.2. Model Results

At first, we tested the CFA model including hedonic and eudaimonic motives and cognitive reappraisal, which was specified as dealing with nondirectional covariance relationships. The results showed that some of the model fit indices did not meet the criteria well (*χ*^2^ _(692)_ = 1218.962, *p* < 0.001, CFI = 0.900, RMSEA = 0.052, SRMR = 0.059). In order to improve the model, we tested the modification indices and allowed the errors of item 1 and item 2 in HEMA-R to correlate in T1 and T2, respectively. The CFA was exerted again, and the model (M1 in Table 2) fitted the data well. We further examined the longitudinal measurement invariance. The metric invariance model (M2) showed a great fit (ΔCFI = 0.001). However, the scalar invariance model (M3) did not have satisfactory indices (ΔCFI = 0.010). According to the modification indices, we released part of the restrain (item 2 in HEMA-R) and the model (M4) indicated a better fit (ΔCFI = 0.008).

**Figure 1:**
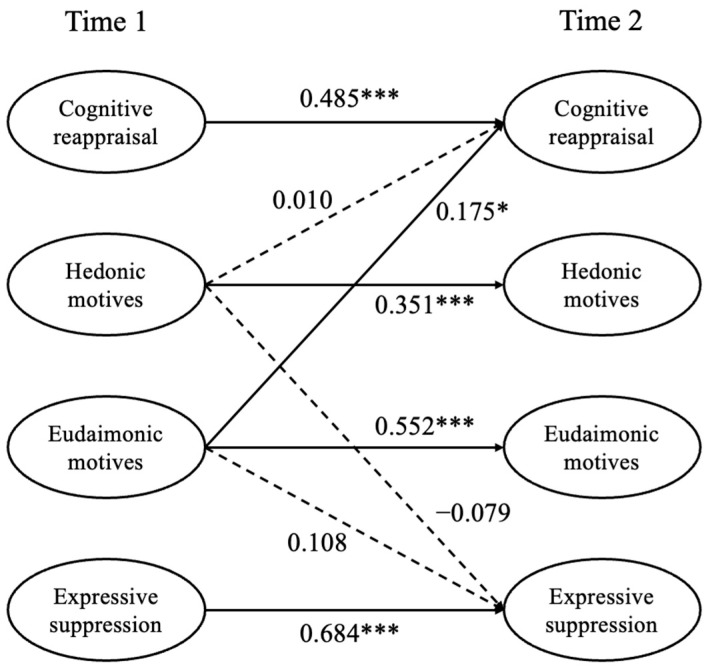
The results of the predictive model (M5). * *p* < 0.05, *** *p* < 0.001.

Under the same constraints, the predictive paths from hedonic and eudaimonic motives to cognitive reappraisal were changed into directional paths. This model (M5) fitted well with the data. All the autoregressive paths were significant (*β*_hedonic motives_ = 0.351, *p* < 0.001; *β*_eudaimonic motives_ = 0.552, *p* < 0.001; *β*_cognitive reappraisal_ = 0.485, *p* < 0.001; *β*_expressive suppression_ = 0.684, *p* < 0.001), indicating that all variables were temporally stable (see Figure 1). In addition, the eudaimonic motives at T1 had a significant positive relationship with cognitive reappraisal at T2 (*β* = 0.175, *p* = 0.029) but not with expressive suppression (*β* = 0.108, *p* > 0.05), while hedonic motivation had no significant relationship with both cognitive reappraisal (*β* = 0.010, *p* > 0.05) and expressive suppression (*β* = −0.079, *p* > 0.05). Furthermore, the results from Model 6 indicated that the link between eudaimonic motives and cognitive reappraisal remained statistically significant, independent of age and gender (*β* = 0.177, *p* = 0.028).

## 4. Discussion

The purpose of this study was to investigate the longitudinal influence of happiness motives on emotion regulation in emerging adults. Overall, the findings indicate that only eudaimonic motives significantly predicted cognitive reappraisal six months later, whereas hedonic motives did not. In contrast, neither motive showed a significant association with expressive suppression. Notably, the relationship between eudaimonic motives and cognitive reappraisal remained stable even after controlling for age and gender. These results provide preliminary evidence regarding the distinct longitudinal role of eudaimonic motives in emotion regulation.

Crucially, the longitudinal findings revealed that eudaimonic motives significantly predicted subsequent cognitive reappraisal, whereas hedonic motives did not. Thus, our first hypothesis regarding the prospective positive links between happiness motives and reappraisal was only partially supported. These results concur with evidence that individuals prefer to use reappraisal for future-oriented and instrumental goals (English et al., 2017; Veilleux et al., 2024). In contrast, cognitive reappraisal may be less effective in reducing negative experiences when individuals prioritize immediate emotional relief, a central feature of hedonic motivation (Sheppes & Meiran, 2007; Y. Wang et al., 2022). This discrepancy may be due to several factors. First, people with hedonic motives aim to change their immediate emotional state and prioritize short-term pleasure rather than enduring benefits (Ortner et al., 2022; Sun et al., 2023; Tamir, 2016). Second, earlier studies have also indicated that hedonic motives tend to yield short-term outcomes, whereas eudaimonic motives are associated with long-term ones, such as well-being (Huta, 2016; Jia et al., 2021; Li et al., 2022). Consistent with this, we only found that hedonic motives were related to cognitive reappraisal at the same time point, without predicting cognitive reappraisal in the long term. Furthermore, as emerging adults actively engage in identity formation and meaning-making, cognitive reappraisal offers a mechanism for meaning-making that supports the “integrated self-functioning” central to eudaimonic pursuits (Curren & Park, 2024). Accordingly, eudaimonic motives may play a more prominent role than hedonic motives in shaping emotion regulation strategies that support self-development and psychological growth in emerging adults.

Furthermore, distinct patterns emerged regarding strategy selection. While we hypothesized that eudaimonic motives would be associated with expressive suppression given its potential instrumental value, the results did not support this prediction. Instead, neither motive showed a significant association with expressive suppression. This null result aligns with recent evidence that engagement-focused strategies like reappraisal are preferentially recruited for long-term emotional goals, whereas suppression reflects a functional mismatch. Regarding hedonic motives, although suppression limits the outward display of emotion, it often fails to alleviate the internal experience of negative affect and can even increase physiological arousal (Dawel et al., 2023; Gross & Levenson, 1997). Recent research indicates that suppression is more closely linked to contra-hedonic motives (e.g., wanting to feel worse or maintain negative affect) rather than the pro-hedonic aim of maximizing pleasure (Shao et al., 2025). Consequently, individuals driven by the pursuit of happiness (hedonic motives) are unlikely to select a strategy that offers little immediate affective relief. Regarding eudaimonic motives, the lack of association with suppression likely stems from the strategy’s conflict with authenticity and social connection—core important components of eudaimonia. While suppression may be employed for specific instrumental goals, such as maintaining superficial social harmony (Han & Kim, 2024; English et al., 2017), its habitual use often impedes the “integrated self-functioning” required for psychological flourishing (Curren & Park, 2024). Unlike reappraisal, which facilitates meaning-making, suppression hinders the processing of emotional information (Caramanica et al., 2023; Richards & Gross, 2000) and creates a barrier to authentic social interaction (Butler et al., 2003), making it functionally incompatible with the growth-oriented nature of eudaimonic pursuits.

There also existed some limitations that should be noted. First, the sample consisted of undergraduates and graduates from a single university, and most participants are females, which may restrict the generalizability of the findings. Future studies should further examine whether these results can remain in other groups with balanced gender, such as teenagers or older adults. Second, although the measures used in our study had satisfactory psychometric properties, reliance on self-reports may introduce bias. To provide a more comprehensive assessment, some other methods should be adopted in future studies, such as peer reports. Third, although the two-wave longitudinal design could identify the temporal relationships of the variables to some extent, it is better to use more wave of data to capture fluctuations and developmental changes in the long run or use experimental designs to clarify the causal relationships.

Despite the aforesaid limitations, the findings of the present study may offer theoretical and practical contributions to the field of emotion regulation. From the perspective of theory, the current study extends the scope of pursuing well-being by confirming the influence of eudaimonic motives on emotion regulation, which underscores the importance of eudaimonic motives. From the perspective of practice, given the beneficial role of eudaimonic motives in promoting cognitive reappraisal, practitioners can design some interventions related to eudaimonic motive and thereby increase the utilization of cognitive reappraisal strategies. Such interventions may further improve individuals’ level of well-being.

## 5. Conclusions

In summary, our findings revealed that eudaimonic motives uniquely predicted cognitive reappraisal six months later, whereas hedonic motives did not show this longitudinal association. In contrast, neither happiness motive was significantly associated with expressive suppression. Importantly, these patterns remained robust even after controlling for age and gender. These results suggest that eudaimonic motives play a distinctive and critical role in fostering adaptive cognitive reappraisal. Therefore, from a practical perspective, these findings highlight the potential utility of interventions designed to cultivate eudaimonic orientations as a means to bolster individuals’ capacity for cognitive reappraisal and overall emotion regulation.

## Figures and Tables

**Table 1:** Descriptive statistics and Pearson correlations.

	*M*	*SD*	Omega	HM1	EM1	CR1	ES1	HM2	EM2	CR2	ES2
HM1	26.533	5.034	0.861	1							
EM1	25.366	4.650	0.808	0.412 ***	1						
CR1	31.146	4.486	0.863	0.279 ***	0.362 ***	1					
ES1	15.244	4.519	0.809	0.069	0.013	0.032	1				
HM2	25.979	4.921	0.895	0.341 ***	0.279 ***	0.188 **	−0.053	1			
EM2	25.453	5.055	0.901	0.305 ***	0.456 ***	0.363 ***	0.014	0.304 ***	1		
CR2	31.181	4.832	0.890	0.269 ***	0.362 ***	0.573 ***	0.051	0.233 ***	0.529 ***	1	
ES2	15.209	4.767	0.838	0.026	0.067	−0.137 *	0.563 ***	−0.050	−0.013	−0.050	1

Note. 1, Time 1; 2, Time 2; CR, cognitive reappraisal; ES, expressive suppression; HM, hedonic motives; EM, eudaimonic motives; * *p* < 0.05; ** *p* < 0.01: *** *p* < 0.001.

**Table 2:** Fit statistics for the model.

								90% CI for RMSEA	
Model	*χ^2^*	*df*	AIC	BIC	CFI	SRMR	RMSEA	Low	Up
M1	1071.815	690	30,841.986	31,464.098	0.927	0.060	0.057	0.039	0.049
M2	1093.889	706	30,835.435	31,398.995	0.926	0.062	0.044	0.039	0.049
M3	1168.203	726	30,873.188	31,363.559	0.916	0.063	0.046	0.041	0.051
M4	1155.690	725	30,861.692	31,355.722	0.918	0.062	0.045	0.041	0.050
M5	1192.189	733	30,887.893	31,352.647	0.913	0.081	0.075	0.042	0.051
M6	1320.827	809	30,893.211	31,372.603	0.905	0.077	0.047	0.042	0.051

Note. AIC = Akaike Information Criterion; BIC = Bayesian Information Criterion; CFI = comparative fit index; SRMR = standardized root mean square residual; RMSEA = root mean square error of approximation; M1 = CFA model; M2 = Metric invariance model; M3 = Scalar invariance model; M4 = Partial scalar invariance model; M5 = Predictive model; M6 = Control model.

## Data Availability

The original contributions presented in this study are included in the article. Further inquiries can be directed to the corresponding author.

## Abbreviations

The following abbreviations are used in this manuscript:

CR	Cognitive Reappraisal
ES	expressive suppression
HMs	hedonic motives
EMs	eudaimonic motives
CFA	confirmatory factor analysis
